# Enhancing mortality prediction in AIDS and disseminated Talaromyces marneffei: the impact of novel inflammatory markers in a nomogram

**DOI:** 10.1099/jmm.0.002066

**Published:** 2025-09-05

**Authors:** Yan Zhang, Xia Zhang, Huaizhong Cui, Kailong Gu, Wenyan Yu, Lingyan He, Yujiao Jin

**Affiliations:** 1 Department of Clinical Laboratory, Hangzhou Xixi Hospital, Hangzhou Sixth People's Hospital, Hangzhou Xixi Hospital Affiliated to Zhejiang Chinese Medical University, Hangzhou, Zhejiang, PR China; 2 Department of Open Laboratory, Hangzhou Xixi Hospital, Hangzhou Sixth People's Hospital, Hangzhou Xixi Hospital Affiliated to Zhejiang Chinese Medical University, Hangzhou, Zhejiang, PR China

**Keywords:** AIDS, bone marrow suppression, C-reactive protein-to-prealbumin ratio, disseminated *Talaromyces marneffei*, procalcitonin-to-albumin ratio

## Abstract

**Introduction.** *Talaromyces marneffei* (TM) is a common opportunistic infection among patients with AIDS, characterized by rapid systemic dissemination and a high mortality rate. Early identification of patients at risk of death is critical to improving clinical outcomes.

**Hypothesis/Gap Statement.** Despite the severity of disseminated TM infection (DPSM), few predictive tools exist to assess mortality risk in affected AIDS patients. A clinical prediction model incorporating novel inflammatory markers may help guide timely intervention.

**Aim.** This study aimed to identify independent risk factors for mortality in AIDS patients with DPSM and to develop and validate a nomogram for individualized risk prediction.

**Methodology.** A retrospective study was conducted on 174 AIDS patients with DPSM and complete clinical data admitted to Hangzhou Xixi Hospital between January 2013 and June 2024. A training cohort of 104 patients was used to identify mortality-related risk factors via logistic regression and to construct a predictive nomogram. The remaining 70 patients constituted a validation cohort to evaluate the model using area under the curve (AUC), decision curve analysis (DCA) and calibration curves.

**Results.** The overall mortality rate was 18.97% (33/174). Effusion, bone marrow suppression, systemic inflammation and malnutrition were significantly associated with fatal outcomes (*P*<0.05). Multivariate logistic regression identified white blood cell count, C-reactive protein-to-prealbumin ratio and procalcitonin-to-albumin ratio as independent risk factors for mortality. The nomogram based on these predictors showed strong discriminative power in both training and validation cohorts (AUC=0.89 and 0.78, respectively). DCA demonstrated the clinical utility and net benefit of the model.

**Conclusion.** This study identified key predictors of mortality in AIDS patients with DPSM and developed a validated nomogram incorporating novel inflammatory markers. The tool offers potential value for individualized risk assessment and clinical decision-making.

## Data Summary

The datasets used to generate the nomograms in this study are included in Supplementary Table S2 of this article.

## Introduction

*Talaromyces marneffei* (TM) is an opportunistic fungal infection that primarily affects populations infected with human immunodeficiency virus (HIV) in tropical and subtropical regions of Asia [1]. Despite the widespread implementation of antiretroviral therapy, this type of invasive fungal disease continues to impose a significant disease burden.

TM primarily invades the reticuloendothelial system of human monocytes and macrophages, leading to two main types of infections: localized and disseminated [2]. Localized infections are rare and typically affect a single organ, such as the lungs, presenting with localized symptoms. In contrast, disseminated infections can involve multiple systems and organs, manifesting as fever, anaemia, weight loss, fatigue, hepatosplenomegaly, lymphadenopathy, cough, sputum production and gastrointestinal discomfort. Some patients may also exhibit central nervous system involvement [1,5]. The recurrence and mortality rates of disseminated TM infection (DPSM) are notably high, with cases of recurrence occurring even years later; mortality rates range from 10% to 30%. Without timely diagnosis and antifungal treatment, the risk of death can increase to as high as 50% [6,8].

Early identification of mortality risk factors in AIDS patients with DPSM is crucial for reducing morbidity and mortality in this population. To our knowledge, while numerous models have been developed to help identify high-risk patients, few have incorporated novel inflammatory markers to predict the prognosis of AIDS patients with DPSM. Consequently, we focused on five core clinical symptoms (fever, cough, fatigue, weight loss and serositis) and laboratory indicators, with a coverage rate of more than 90% and prognostic stratification value [1,3, 7, 8]. We have established a simple and practical nomogram model that includes these novel inflammatory markers, providing a basis for early clinical prevention and treatment of AIDS patients with DPSM.

## Methods

### Patients and ethics

This retrospective study included hospitalized patients diagnosed with AIDS and DPSM at Hangzhou Xixi Hospital from January 2013 to December 2024. Hangzhou Xixi Hospital, located in Hangzhou, Zhejiang Province, is a government-designated tertiary teaching hospital specializing in complex and challenging AIDS cases. The inclusion criteria were as follows: (1) age ≥18 years, (2) confirmed HIV infection as defined by the Centers for Disease Control and Prevention [9] and (3) positive culture for TM from patient specimens (including blood, bone marrow, sputum, bronchoalveolar lavage fluid and lymph nodes) was performed. Clinical specimens were cultured on Sabouraud dextrose agar at both 25 and 37 °C. To minimize bias, the exclusion criteria included the following: (1) with incomplete data, (2) age <18 years, (3) without HIV infection and (4) other immunodeficiencies (including malignancies and congenital immunodeficiencies).

**Fig. 1.**
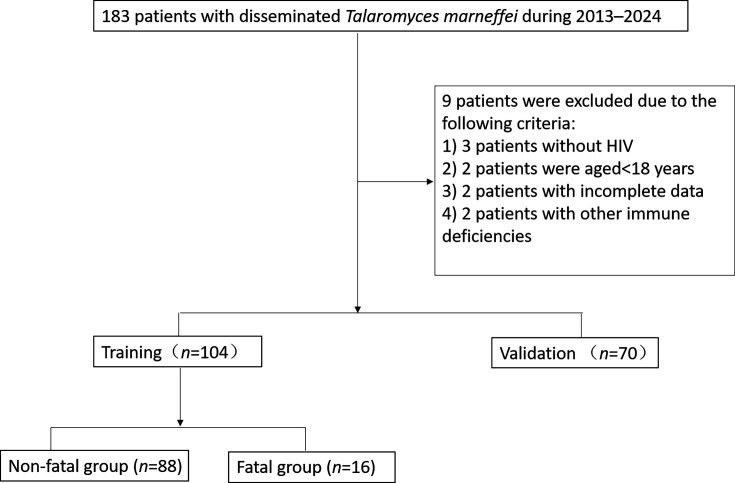
Flowchart of AIDS/DPSM patient enrolment.

The flow chart of the study participants is shown in Fig. 1. Patients were classified into fatal and non-fatal groups on the basis of their prognosis. The non-fatal group comprised 141 patients who were either cured or showed improvements after treatment, whereas the fatal group included 33 patients who died during hospitalization or were ultimately discharged. The patients were randomly divided into a training group (104 cases) and a validation group (70 cases) in a ratio of 6 : 4. The study protocol was approved by the Ethics Committee of Hangzhou Xixi Hospital.

### Clinical variables

Data extracted from medical records included demographic information (sex and age), clinical characteristics (symptoms and signs) and laboratory results at admission [white blood cell (WBC) count, red blood cell (RBC) count, haemoglobin (HB), platelet (PLT) count, C-reactive protein (CRP), CD4 cell count, CD8 cell count, CD4/CD8 ratio, prealbumin (PA), albumin (ALB), procalcitonin (PCT), HIV RNA, C-reactive protein-to-prealbumin ratio (CPAR), C-reactive protein-to-albumin ratio (CAR), procalcitonin-to-albumin ratio (PAR) and bone marrow smear results].

To address the potential bias in this retrospective cohort study, we implemented several strategies. Selection bias was minimized by including all eligible HIV/TM co-infected patients admitted to Hangzhou Xixi Hospital between January 2013 and December 2024. Information bias was reduced by using standardized data collection methods and verifying clinical and laboratory data through hospital records. We also preprocessed the data, removed some real data and checked the data for outliers. Patient data were carefully reviewed item by item. To ensure accuracy, all information was rigorously cross-checked with medical records by two researchers. All research laboratories followed standardized and certified procedures to ensure their quality and reliability. Serositis is defined as involvement of the pleural space, resulting in pleural effusion. The PLT-producing function of megakaryocytes was evaluated according to the number of PLT-producing megakaryocytes: less than 3 were defined as very poor function, 4–5 were considered poor function, 6–7 were considered fair function and more than 8 were considered good function.

The percentage of missing data for continuous variables was less than 20% and was replaced by the mean or median in the analysis. Variables with missing values exceeding 20% were not included. This study analysed the characteristics of patients with AIDS and DPSM and subsequently evaluated the risk factors for mortality and established a mortality prediction model. The primary outcome was death, while the exposure factors included clinical characteristics and initial laboratory indicators at admission.

### Statistics

Mean imputation was used for variables with missing data rates below 20%, including CD4, CD8, CD4/CD8, ALB, PA, CRP, CPAR, CAR, PAR and PCT. Normality was assessed by Shapiro–Wilk tests (*P*>0.05 threshold) with visual inspection of histograms. Data conforming to a normal distribution are expressed as the means±sd and were compared via t-tests. For nonnormally distributed data, the Mann–Whitney U test was employed. Categorical variables are presented as percentages, with comparisons made via the χ² test or Fisher’s exact test. In the training cohort, both univariate and multivariate logistic regression analyses were conducted, and a stepwise method was applied to identify independent risk factors for mortality. We controlled for confounding factors through multivariate logistic regression. On the basis of these independent risk factors, a nomogram was constructed to predict the mortality probability for each AIDS patient with DPSM. Internal validation of the nomogram was performed in the validation cohort. The discrimination ability of the nomogram was assessed via the area under the receiver operating characteristic curve (ROC AUC) and decision curve analysis (DCA). The calibration curves were used to compare the actual results and the nomogram-predicted probabilities. Considering the limited number of death events in the training cohort, a sensitivity analysis was conducted to mitigate the estimation bias associated with traditional logistic regression in the context of small sample sizes and sparse data. The Firth penalized likelihood method was utilized, implemented through the logistf package in R, to evaluate the robustness of the primary analysis results regarding the selected risk factors. A *P* value of <0.05 was considered statistically significant.

## Results

### Demographic characteristics

This study included a total of 174 patients, including 157 males (90.23%). The median age was 35 years, with a mortality rate of 18.97% (33/174). Among the patients, 127 (72.99%) were under 45 years of age, 40 (22.99%) were between 45 and 60 years and 7 (4.02%) were over 60 years. A total of 104 patients composed the training cohort, whereas 70 patients composed the validation cohort.

### Clinical characteristics

The most prevalent symptoms and signs included fever (160 cases, 91.95%), cough (123 cases, 70.69%), fatigue (90 cases, 51.72%), serositis (82 cases, 47.13%), weight loss (65 cases, 37.36%) and positive HIV RNA results (135 cases, 88.82%). The characteristics of the patients in both the training and validation cohorts are detailed in Table 1. Table 2 presents the characteristics of patients in the fatal and non-fatal groups within the validation cohort.

**Table 1.** Baseline data of patients in the training cohort and validation cohort

Variable	Total (*n*=174)	Validation (*n*=70)	Training (*n*=104)	*P*
Sex, *n* (%)				0.34
Male	157 (90.23)	65 (92.86)	92 (88.46)	-
Female	17 (9.77)	5 (7.14)	12 (11.54)	-
Age (years)	35.00 (29.00, 46.00)	34.00 (28.25, 47.50)	35.00 (30.00, 46.00)	0.58
Fever, *n* (%)	160 (91.95)	64 (91.43)	96 (92.31)	0.83
Cough, *n* (%)	123 (70.69)	55 (78.57)	68 (65.38)	0.06
Fatigue, *n* (%)	90 (51.72)	32 (45.71)	58 (55.77)	0.19
Weight loss, *n* (%)	65 (37.36)	23 (32.86)	42 (40.38)	0.31
Serositis, *n* (%)	82 (47.13)	31 (44.29)	51 (49.04)	0.54
HIV RNA, *n* (%)	135 (88.82)	57 (87.69)	78 (89.66)	0.70
WBC (10^9^/l)	3.73 (2.56, 5.29)	3.56 (2.32, 5.07)	3.95 (2.73, 5.40)	0.27
RBC (10^12^/l)	3.42±0.76	3.35±0.71	3.47±0.78	0.32
HB (g l^−1^)	97.00 (83.25, 114.50)	95.00 (83.00, 109.75)	99.50 (84.75, 115.00)	0.36
PLT (10 ^9^/l)	110.50 (64.00, 181.00)	108.50 (61.00, 164.75)	113.50 (64.75, 190.75)	0.51
CRP (mg l^−1^)	60.00 (30.91, 96.17)	67.89 (38.97, 119.00)	56.69 (28.90, 90.00)	0.10
CD4 count (cells/µl)	9.00 (3.00, 23.00)	7.50 (3.00, 20.25)	10.00 (4.00, 23.00)	0.33
CD8 count (cells/µl)	188.00 (115.50, 321.50)	188.00 (124.25, 284.25)	191.00 (115.00, 327.50)	0.83
CD4/CD8 ratio	0.05 (0.02, 0.09)	0.04 (0.02, 0.09)	0.05 (0.03, 0.10)	0.18
PA (mg l^−1^)	58.00 (25.75, 104.00)	48.00 (23.00, 84.00)	69.00 (27.00, 121.50)	0.04
CPAR×10^2^	100.80 (39.84, 285.00)	140.03 (46.33, 420.45)	84.52 (23.29, 259.67)	0.09
ALB (g l^−1^)	25.70 (22.55, 30.33)	24.50 (20.50, 28.80)	26.70 (23.60, 31.55)	0.01
CAR×10^−5^	220.12 (115.02, 395.21)	271.47 (147.08, 483.53)	193.43 (108.30, 357.95)	0.05
PCT (ng ml^−1^)	0.64 (0.22, 2.24)	0.70 (0.31, 3.83)	0.50 (0.15, 1.96)	0.04
PAR×10^−8^	2.49 (0.71, 9.55)	3.19 (1.15, 14.61)	1.94 (0.54, 7.69)	0.03

CD4, CD4+ T lymphocytes; CD8, CD8+ T lymphocytes.

**Table 2.** Baseline data of patients in the fatal group and non-fatal group of the training cohort

Variable	Total (*n*=104)	Non-fatal (*n*=88)	Fatal (*n*=16)	*P*
Age (years)	35.00 (30.00, 46.00)	35.00 (29.00, 44.00)	44.50 (30.75, 52.25)	0.24
Sex, *n* (%)				1.00
Male	92 (88.46)	78 (88.64)	14 (87.50)	-
Female	12 (11.54)	10 (11.36)	2 (12.50)	-
Fever, *n* (%)	96 (92.31)	80 (90.91)	16 (100.00)	0.46
Cough, *n* (%)	68 (65.38)	60 (68.18)	8 (50.00)	0.16
Fatigue, *n* (%)	58 (55.77)	49 (55.68)	9 (56.25)	0.97
Weight loss, *n* (%)	42 (40.38)	32 (36.36)	10 (62.50)	0.05
Serositis, *n* (%)	51 (49.04)	39 (44.32)	12 (75.00)	0.02
HIV RNA, *n* (%)	78 (89.66)	67 (89.33)	11 (91.67)	1.00
WBC (10^9^/l)	3.95 (2.73, 5.40)	3.73 (2.55, 5.19)	4.81 (3.58, 10.50)	0.01
RBC (10^12^/l)	3.56 (2.99, 4.02)	3.62 (3.01, 4.08)	3.21 (2.60, 3.68)	0.09
HB (g l^−1^)	99.50 (84.75, 115.00)	101.50 (87.00, 116.00)	88.00 (61.25, 105.50)	0.02
PLT (10^9^/l)	113.50 (64.75, 190.75)	123.50 (77.50, 207.25)	43.50 (22.00, 81.75)	<0.01
CRP (mg l^−1^)	56.69 (28.90, 90.00)	54.94 (27.93, 84.94)	76.96 (53.00, 147.23)	0.04
CD4 count (cells/µl)	10.00 (4.00, 23.00)	10.00 (4.00, 23.00)	8.00 (2.50, 29.00)	0.65
CD8 count (cells/µl)	191.00 (115.00, 327.50)	197.00 (116.00, 325.00)	142.00 (86.50, 309.25)	0.55
CD4/CD8 ratio	0.05 (0.03, 0.10)	0.05 (0.02, 0.10)	0.06 (0.04, 0.07)	0.75
PA (mg l^−1^)	69.00 (27.00, 121.50)	78.50 (45.75, 128.00)	23.00 (15.00, 35.00)	<0.01
CPAR×10^2^	84.52 (23.29, 259.67)	75.64 (22.61, 190.60)	344.06 (134.33, 1010.28)	0.01
ALB (g l^−1^)	26.70 (23.60, 31.55)	27.25 (23.90, 32.12)	22.60 (20.80, 26.15)	<0.01
CAR×10^−5^	193.43 (108.30, 357.95)	192.39 (106.32, 341.12)	331.82 (172.64, 692.22)	0.04
PCT (ng ml^−1^)	0.50 (0.15, 1.96)	0.39 (0.14, 1.27)	7.87 (0.65, 28.85)	<0.01
PAR×10^−8^	1.94 (0.54, 7.69)	1.32 (0.45, 4.92)	34.40 (2.70, 130.10)	<0.01

CD4, CD4+ T lymphocytes; CD8, CD8+ T lymphocytes.

### Laboratory investigations

Among the patients, 172 had positive blood cultures, 22 had positive bone marrow cultures, 12 had positive serous cavity effusion cultures, 12 had positive bronchoalveolar lavage cultures, 30 had positive sputum cultures, 15 had positive tissue cultures and 11 had positive throat swabs.

A comprehensive analysis of 15 laboratory parameters was conducted for both the fatal and non-fatal groups, and the median levels were compared. In the fatal group, the WBC, CRP, CPAR, CAR, PCT and PAR values were significantly greater than those in the non-fatal group (*P* values of 0.01, 0.04, 0.01, 0.04, <0.01 and <0.01, respectively). Conversely, the levels of HB, PLT, PA and ALB in the non-fatal group were significantly lower than those in the fatal group (*P* values of 0.02, <0.01, <0.01 and <0.01, respectively). The detailed results can be found in Table 2.

Bone marrow smear was performed in 31 of the 174 patients, and bone marrow culture was performed in 29 patients. The assessment revealed that 7 patients tested positive for TM, whereas 15 patients presented with haematophagous cells. Notably, 22 patients (70.97%) displayed a low or absent quantity of myeloid cells, indicating overall poor haematopoietic function. Myeloid hyperplasia was observed in 26 patients (83.87%), and erythroid hyperplasia was noted in 23 patients (74.19%). The average number of megakaryocytes was 30 (ranging from 10 to 106). Among these, six patients demonstrated good megakaryocyte function, three had fair function, four had poor function and one had very poor function, while nine patients were not assessed due to a low number of megakaryocytes. Pathological haematopoiesis to varying degrees was present in 21 patients (67.74%), affecting myeloid, erythroid and megakaryocyte lineages. The myeloid pathological features included megaloblastic changes, ringed nuclei, binucleation and abnormal nuclear lobulation. Erythroid pathological features included binucleation, multinucleation, flower-shaped nuclei, nuclear bridging, nuclear budding and megaloblastic changes. The pathological features of megakaryocytes include multinucleated megakaryocytes, hyperlobulated megakaryocytes and small or hypoplastic megakaryocytes.

### Independent risk factors for mortality in AIDS patients with DPSM and the nomogram

**Fig. 2.**
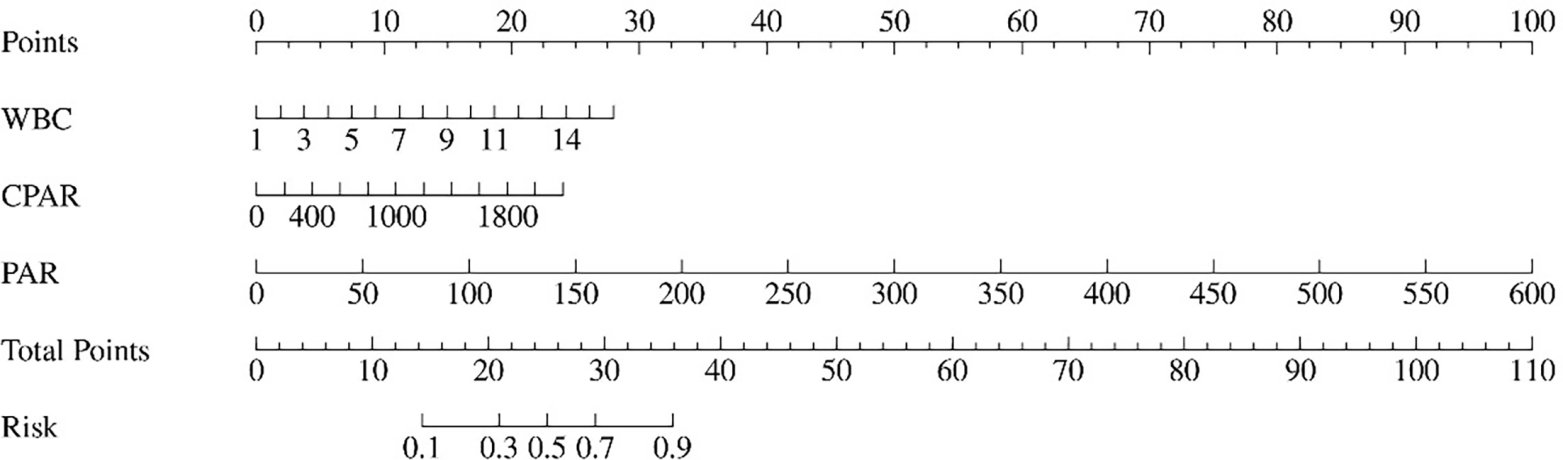
Diagnostic value of different markers in predicting death.

Statistically significant indicators identified in the univariate analysis were included in the binary logistic regression analysis. The results indicated that the WBC count [*β*=0.38; odds ratio (OR): 1.46; 95 % confidence interval (CI): 1.13–1.89; *P*<0.01], CPAR (*β*=0.01; OR: 1.01; 95 % CI: 1.01–1.01; *P*=0.04) and PAR (*β*=0.03; OR: 1.03; 95 % CI: 1.01–1.07; *P*=0.02) were independent risk factors for mortality in AIDS patients with DPSM (Table 3). The predictive probability model for death in these patients was determined as logit (*P*)=−5.484+0.38×WBC+0.01×CPAR+0.03×PAR. A nomogram was constructed on the basis of these independent risk factors (Fig. 2). Each factor is assigned a score in the scoring table. By summing the scores of each factor, a total score is obtained on the ‘total score line’. A vertical line drawn from this point intersects the mortality probability line, indicating the estimated mortality probability for each AIDS patient with DPSM. For instance, a patient with WBC=5×10⁹/l, CPAR=150 and PAR=10 would receive ~10 points for WBC, 2 points for CPAR and 1 point for PAR, resulting in a total score of 13, which corresponds to an estimated mortality risk of 14%.

**Table 3.** Results of univariate and multivariate logistic regression

Variable	Univariate					Multivariate				
	*β*	S.E	*Z*	*P*	OR (95% CI)	*β*	S.E	*Z*	*P*	OR (95% CI)
Sex (Female)	0.11	0.83	0.13	0.90	1.11 (0.22–5.64)	-	-	-	-	-
Fever	0.67	1.08	0.62	0.534	1.96 (0.24–16.24)	-	-	-	-	-
Cough	−0.76	0.55	−1.39	0.17	0.47 (0.16–1.37)	-	-	-	-	-
Fatigue	0.02	0.55	0.04	0.97	1.02 (0.35–2.99)	-	-	-	-	-
Weight loss	1.07	0.56	1.91	0.06	2.92 (0.97–8.77)	-	-	-	-	-
Serositis	1.33	0.62	2.15	0.03	3.77 (1.13–12.60)	-	-	-	-	-
HIV RNA	0.27	1.11	0.25	0.81	1.31 (0.15–11.55)	-	-	-	-	-
Age	0.03	0.02	1.30	0.19	1.03 (0.98–1.08)	-	-	-	-	-
WBC	0.23	0.08	2.77	<0.01	1.26 (1.07–1.49)	0.38	0.13	2.90	<0.01	1.46 (1.13–1.89)
RBC	−0.71	0.34	−2.08	0.04	0.49 (0.25–0.96)	-	-	-	-	-
HB	−0.03	0.01	−2.52	0.01	0.97 (0.95–0.99)	-	-	-	-	-
PLT	−0.02	0.01	−2.87	<0.01	0.98 (0.97–0.99)	-	-	-	-	-
CRP	0.02	0.01	2.57	0.01	1.02 (1.01–1.03)	-	-	-	-	-
CD4	−0.00	0.01	−0.15	0.88	1.00 (0.98–1.02)	-	-	-	-	-
CD8	0.00	0.00	0.77	0.44	1.00 (1.00–1.00)	-	-	-	-	-
CD4/CD8 ratio	1.88	1.76	1.07	0.29	6.55 (0.21–207.35)	-	-	-	-	-
PA	−0.02	0.01	−2.28	0.02	0.98 (0.97–0.99)	-	-	-	-	-
CPAR	0.01	0.00	2.94	<0.01	1.01 (1.01–1.01)	0.01	0.00	2.06	0.04	1.01 (1.01–1.01)
ALB	−0.17	0.06	−2.81	<0.01	0.84 (0.75–0.95)	-	-	-	-	-
CAR	0.01	0.00	2.94	<0.01	1.01 (1.01–1.01)	-	-	-	-	-
PCT	0.11	0.04	2.92	<0.01	1.11 (1.04–1.20)	-	-	-	-	-
PAR	0.04	0.01	2.90	<0.01	1.04 (1.01–1.06)	0.03	0.02	2.25	0.02	1.03 (1.01–1.07)

### Calibration and clinical utility

**Fig. 3.**
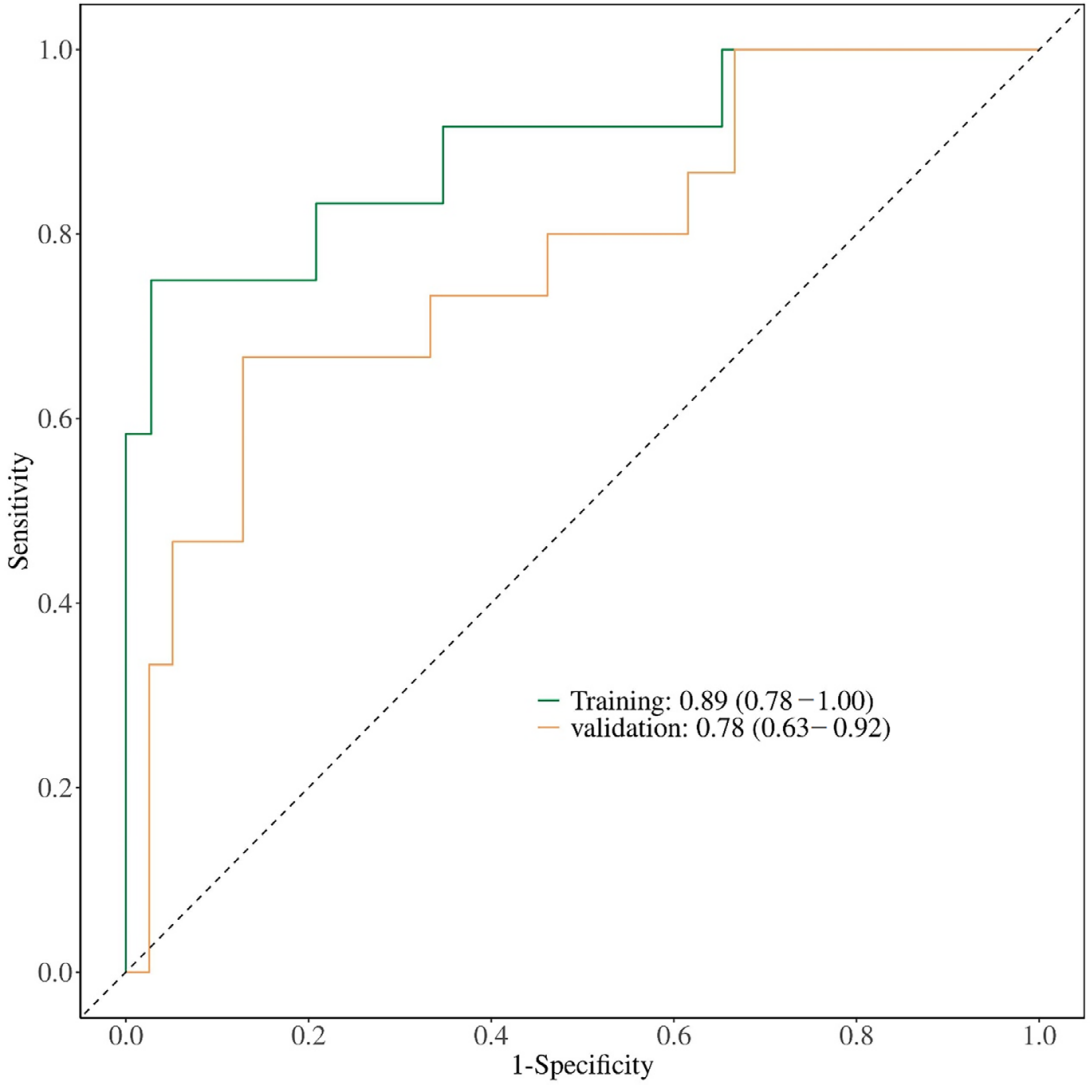
ROC curves of the training cohort and validation cohort.

**Fig. 4.**
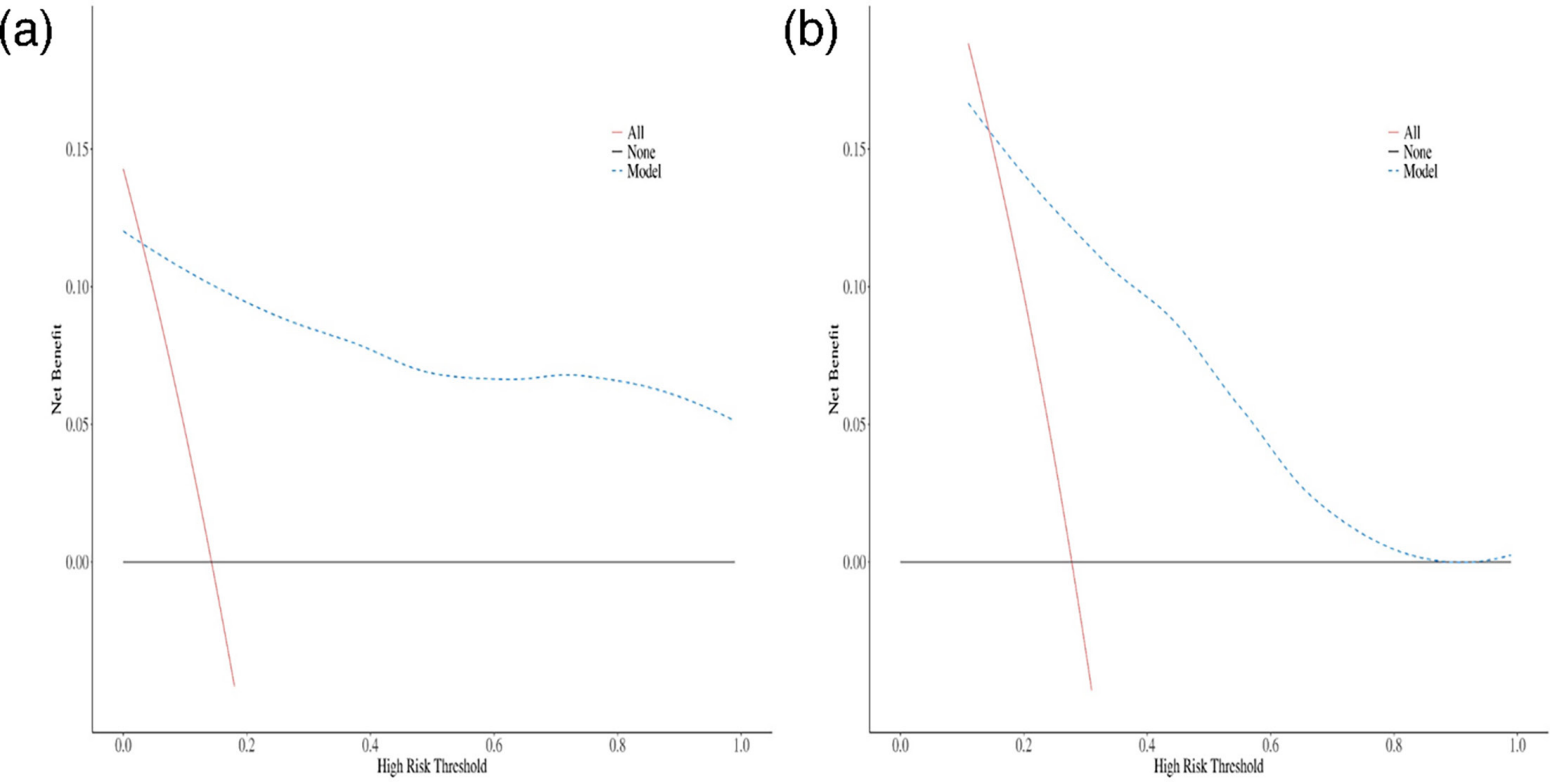
DCA of training cohort (**a**) and validation cohort (**b**).

**Fig. 5.**
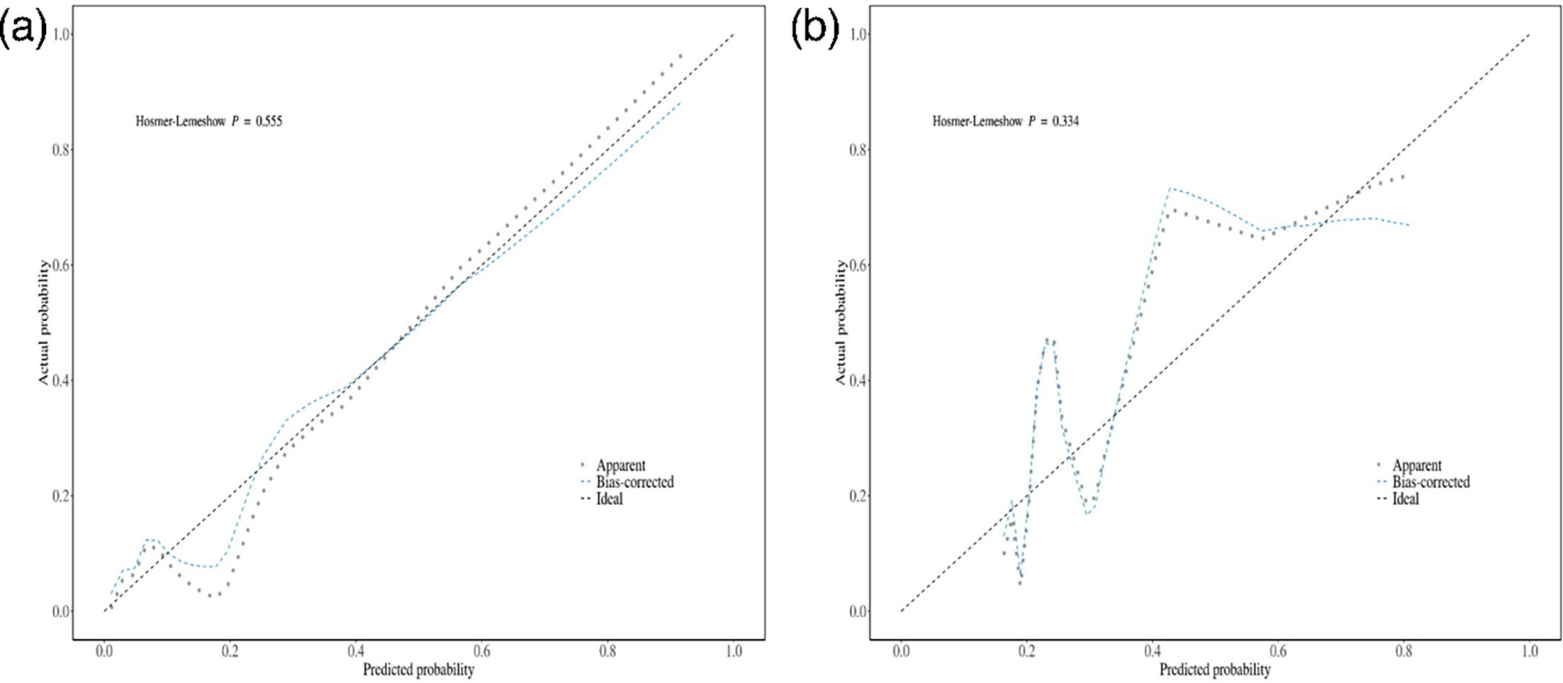
The calibration curve of the training cohort (**a**) and validation cohort (**b**).

The predictive value of the nomogram was evaluated via the AUC (Table 4, Fig. 3). For Fig. 3, the x-axis represents ‘1-Specificity’ and the y-axis represents ‘Sensitivity’. The ROC curve for the training cohort yielded an AUC of 0.89 (95 % CI: 0.78–1.00), whereas the ROC curve for the validation cohort had an AUC of 0.78 (95 % CI: 0.63–0.92), indicating high predictive capability. The optimal cutoff value for the nomogram in the training cohort was 0.312, with a sensitivity and specificity of 0.97 and 0.75, respectively. In the validation cohort, the optimal cutoff value remained 0.312, with a sensitivity of 0.95 and specificity of 0.47. We evaluated the clinical utility of the nomogram model by DCA (Fig. 4). For Fig. 4, the x-axis represents the ‘Threshold Probability’ and the y-axis represents the ‘Net Benefit’. In the validation cohort, our nomogram model provided a net benefit greater than that of the ‘all’ and ‘none’ strategies within the threshold probability range (15 %–85 %), indicating that the model has practical value (Fig. 4b). The calibration curve is shown in Fig. 5. The *P* values are all greater than 0.05. In the training cohort, the calibration curve showed excellent agreement between predicted probabilities and actual outcomes, indicating good model fit (Fig. 5a). In the validation cohort, the calibration curve exhibited moderate fluctuations but remained generally aligned with the ideal reference line (Fig. 5b). This variation is likely attributable to the smaller sample size and inherent variability in the validation dataset. Despite this, the model maintained acceptable calibration performance.

**Table 4.** Confusion matrix for training and validation cohorts

Data	AUC (95% CI)	Accuracy (95% CI)	Sensitivity (95% CI)	Specificity (95% CI)	PPV (95% CI)	NPV (95% CI)	Cutoff
Training	0.89 (0.78–1.00)	0.94 (0.87–0.98)	0.97 (0.93–1.00)	0.75 (0.51–0.99)	0.96 (0.91–1.00)	0.82 (0.59–1.00)	0.312
Validation	0.78 (0.63–0.92)	0.81 (0.69–0.91)	0.95 (0.88–1.00)	0.47 (0.21–0.72)	0.82 (0.71–0.93)	0.78 (0.51–1.00)	0.312

NPV, Negative Predictive Value; PPV, Positive Predictive Value.

## Discussion

In the context of the HIV epidemic, TM infection poses a significant public health challenge. In this study, we analysed the mortality and risk factors for death of AIDS patients with DPSM in Hangzhou, Zhejiang Province, China. Although previous studies have explored the prognostic value of CRP and PCT in AIDS-related sepsis [10,11], our study is the first to examine the CPAR and PAR as composite markers. These ratios may better reflect the interplay between systemic inflammation and nutritional status, offering additional prognostic value.

In this study, the most common clinical symptoms in AIDS patients with DPSM were fever, cough and fatigue, which is consistent with previous research [12,13]. Notably, we observed significant differences in serositis between the non-fatal and fatal groups. Some studies suggest that serositis is associated with patient prognosis [7]; when the TM invades serous membranes, it can lead to misdiagnosis as tuberculosis, resulting in increased mortality rates [14]. These findings are rare and warrant attention.

Our patients exhibited significant laboratory abnormalities, including bone marrow suppression, systemic inflammation and malnutrition. Bone marrow morphology examination has been recognized as a valuable tool for the early diagnosis of DPSM infection [15]. In this cohort, haematopoietic suppression was frequently observed, as evidenced by decreased HB and PLT levels, potentially attributable to the pathogen’s propensity to invade vascular structures and disrupt bone marrow function [16]. Among the 31 patients who underwent bone marrow examination, 70.97% showed reduced or absent myeloid cells, indicating impaired haematopoiesis. Additionally, 48.39% of these patients exhibited haemophagocytic features, consistent with the findings reported by Zeng *et al*. [17]. However, due to the limited sample size and lack of standardized bone marrow assessments, these findings were not incorporated into the final predictive model to avoid potential bias. Further studies with larger cohorts and standardized protocols are warranted to elucidate the prognostic significance of haematopoietic suppression in DPSM.

Multivariate analysis revealed that novel inflammatory markers (CPAR and PAR) are independent risk factors for mortality in patients. CRP is an acute-phase reactant synthesized by hepatocytes and serves as a nonspecific clinical marker for systemic inflammation. PCT levels are reliable indicators of fungal infection, significantly increasing and remaining elevated in cases of infection and trauma. CRP and PCT levels can reflect the severity of a patient’s condition and are noteworthy predictors of disease mortality [10,11]. Both ALB and PA are synthesized by hepatocytes, with ALB being the most abundant protein in plasma, serving as an indicator of individual nutritional status. PA reflects liver cell damage earlier than does ALB and may be present at abnormal levels in various conditions, such as acute inflammation and malignancies. The CPAR and PAR have been extensively studied as novel inflammatory markers, indicating that inflammation and malnutrition serve as predictors of disease progression [18,22]. The relationship between inflammation and malnutrition is complex, as inflammation can lead to malnutrition, which in turn exacerbates inflammation. Our study identified inflammation and malnutrition as independent risk factors for mortality in AIDS patients with DPSM. Early nutritional supplements, anti-inflammatory medications and antiviral treatments are crucial to prevent disease deterioration in these patients. Given the limited sample size and relatively few deaths in this study, Firth’s penalized logistic regression was employed to conduct a sensitivity analysis of the primary risk factors (Table S1, available in the online Supplementary Material). The results indicated that WBC and PAR remained independent risk factors for mortality after adjustment, aligning closely with the main analysis conclusions. Notably, while CPAR was statistically significant in the standard logistic regression, it did not achieve significance in the Firth regression, suggesting that its association with mortality risk may be influenced by sample size and sparsity. Therefore, the role of CPAR warrants further validation in a larger sample study.

This research constructed and validated a nomogram to predict the mortality probability for each AIDS patient with DPSM by combining three predictive factors. Furthermore, our model indicates that the novel inflammatory markers CPAR and PAR have significant predictive value for mortality risk. Our nomogram may help estimate the mortality probability for each AIDS patient with DPSM, potentially improving patient outcomes, suggesting that clinicians should closely monitor the dynamic changes in these inflammatory indicators.

However, our study has several limitations. First, this study is subject to the inherent limitations of retrospective chart reviews, including potential selection bias, information bias and incomplete documentation. Although we implemented strict inclusion criteria and manual data validation, we acknowledge that such designs are less robust for establishing causality. The methodological considerations and limitations of retrospective analyses have been well discussed in previous literature [23,25], and we have taken steps to mitigate these biases where possible. The nomogram developed in this study requires prospective validation in larger, independent cohorts to confirm its clinical utility and generalizability. Second, although this study excluded individuals with malignancies to focus on the independent effect of DPSM, the synergistic pathogenicity of the two in AIDS cannot be ignored. Future prospective cohort studies are needed to compare the differences between patients with DPSM alone and patients with DPSM combined with malignancies in order to optimize integrated treatment strategies. Furthermore, another limitation of this study is the lack of detailed data on antifungal treatment regimens, including drug selection, timing of initiation and adherence. These factors may influence patient outcomes and should be considered in future prospective research. Finally, the calibration curve of the validation set shows significant fluctuations. While the H-L test *P* value is greater than 0.05, the sample size of this validation set is relatively small (*N*=70), which limits the reliability of the H-L test in such contexts. Therefore, we cannot conclude that the model calibration is robust in the validation set, necessitating further testing in a larger validation cohort in the future.

## Conclusion

Overall, this retrospective cohort study identified WBC, CPAR and PAR as independent risk factors for mortality in patients with AIDS patients with DPSM. The value of predictive models for assessing prognosis in patients, utilizing both existing data and novel inflammatory markers, is increasingly recognized as a cost-effective approach to support clinical decision-making. The nomogram that incorporates these factors demonstrated superior predictive capabilities for adverse outcomes. This tool can guide clinical decision-making in AIDS patients with DPSM, facilitating timely diagnosis and treatment and ultimately enhancing patient outcomes.

## Supplementary material

10.1099/jmm.0.002066 Uncited Table S1.

10.1099/jmm.0.002066 Uncited Supplementary Data Sheet 1.

## Abbreviations

ALB: albumin
AUC: area under the curve
CAR: C-reactive protein-to-albumin ratio
CI: confidence interval
CPAR: C-reactive protein-to-prealbumin ratio
CRP: C-reactive protein
DCA: decision curve analysis
HB: haemoglobin
OR: odds ratio
PA: prealbumin
PAR: procalcitonin-to-albumin ratio
PCT: procalcitonin
PLT: platelet
RBC: red blood cell
ROC: receiver operating characteristic
TM: *Talaromyces marneffei*
WBC: white blood cell
