# Supplementary material for: Enhancing mortality prediction in AIDS and disseminated Talaromyces marneffei: the impact of novel inflammatory markers in a nomogram
Source: J Med Microbiol. 2025 Sep 5;74(9):002066. doi: 10.1099/jmm.0.002066 (PMC12413300; doi:10.1099/jmm.0.002066)
Supplement: Uncited Table S1. [file jmm-74-02066-s001.pdf]

S1 Results of Firth's penalized logistic regression

Variables	β	OR (95% CI)	*P*
WBC	0.160	1.17 (1.03–1.34)	0.015
CPAR	0.0003	1.0003 (0.9998–1.0009)	0.236
PAR	0.0090	1.009 (1.003–1.022)	0.002

Table Notes: OR is odds ratio and CI is 95% confidence interval.
